# Gut flora profiling and fecal metabolite composition of colorectal cancer patients and healthy individuals

**DOI:** 10.3892/etm.2022.11175

**Published:** 2022-01-31

**Authors:** Xiaoxue Wang, Jianping Wang, Benqiang Rao, Li Deng

Exp Ther Med 13:2848–2854, 2017; DOI: 10.3892/etm.2017.4367

Following the publication of this article, an interested reader drew to our attention that data and figures (particularly relating to Figs. 1 and 3) bore some striking and unexpected similarities with figures in the following article that was published in PLoS One [Weir TL, Manter DK, Sheflin AM, Barnett BA, Heuberger AL and Ryan EP: Stool microbiome and metabolome differences between colorectal cancer patients and healthy adults. PLoS One. 8: e70803, 2013].

After having conducted an internal investigation, the Editor of *Experimental and Therapeutic Medicine* agrees that the similarities among the abovementioned figures and those that appeared in the PLoS One article were too extensive that they may have been purely coincidental. The authors were contacted about this issue and were asked to comment on the apparent similarities, but they failed to respond to our queries in this regard. Therefore, on the grounds of a lack of confidence in the presented data, the Editor of *Experimental and Therapeutic Medicine* has decided that the article should be retracted from the publication. The Editor sincerely apologizes to the authors of the PLoS article and to the readership for the inconvenience caused. We also thank the reader for bringing this matter to our attention.

